# Accumulation Function for the Ideal Air-Standard Brayton Cycle Based on Serrin’s Thermodynamics

**DOI:** 10.3390/e27121228

**Published:** 2025-12-04

**Authors:** Vidal Aquiles de Jesus Sanchez-Sanchez, Pedro Quinto Diez

**Affiliations:** Escuela Superior de Ingeniería Mecánica y Eléctrica Unidad Zacatenco, Instituto Politécnico Nacional, Mexico City 07738, Mexico; pquintod@ipn.mx

**Keywords:** thermodynamic work, temperature, accumulation function, ideal Brayton cycle

## Abstract

Serrin’s works provided a new perspective on classical thermodynamics through his statements of the first law and the accumulation function, and of the second law and the accumulation theorem, as well as the subsequent result by Huilgol that the work accomplished in a thermal cycle implies an inequality where the important temperatures of the thermal cycle and an integral similar to that of Clausius appears. Based on these pioneering works, explicit forms of the accumulation function have been derived for the Otto, Diesel, Stirling and Ericsson cycles. In this paper, a more straightforward derivation than that made by Huilgol is presented to obtain the inequality for the work accomplished in a cycle, following the theoretical framework of Serrin and Huilgol, and explicitly introducing that the temperature ranges in which the system exchanges heat are finite. This paper clearly shows the natural physical fact that heat exchange processes in a system have two defined extreme temperatures, corresponding to the beginning and end of the process, which can be equal in the isothermal limiting case. The derivation of the accumulation function for the ideal air-standard Brayton cycle is provided for the first time, extending Serrin’s thermodynamic framework, where the temperature constraints of the adiabatic compression and expansion processes under which it operates are analyzed. Finally, a practical example is included to illustrate the behavior of the accumulation function of the ideal air-standard Brayton cycle.

## 1. Introduction

Until 1978, classical thermodynamics had been developed through the contributions of Sadi Carnot (1796–1832), Rudolf Clausius (1822–1888), William Thomson (Lord Kelvin, 1824–1907), Max Planck (1858–1947), and James Joule (1818–1889) regarding the first and second laws of thermodynamics [1].

Based on experimental observations, the first law of classical thermodynamics states that energy cannot be created or destroyed during a process; it can only change forms [1,2,3,4].

A conventional thermodynamic analysis of an energy conversion system involves the application of the first law of thermodynamics, which is also known as energy analysis [2]. This analysis shows how internal energy, heat, and work are related during the processes occurring within the system. Since energy is a conserved property and no process has ever been observed to violate the first law, it is reasonable to conclude that any process occurring in nature must comply with the first law for it to take place. However, satisfying the first law does not guarantee that the process can occur. This is because processes advance in a specific direction and not in the reverse direction. The first law imposes no restrictions on the direction in which a process occurs [1]. This insufficiency of the first law to determine whether a process can occur is resolved by introducing another general principle: the second law of thermodynamics. In the literature, there are several standard forms of the second law, which are believed to be equivalent and are attributed to Clausius, Kelvin, and Planck [5,6,7,8].

The second law of thermodynamics allows for the identification of the direction in which natural processes occur, defines the degree of perfection of thermodynamic processes, and can be used to quantify this level of perfection, effectively pointing the way to optimize efficiency losses. It introduces the new state function called entropy and asserts that energy possesses quality as well as quantity [2]. Therefore, a process cannot occur unless it satisfies both the first and second laws of thermodynamics.

However, starting in 1979, Serrin’s research in thermodynamics provided new insights into the meaning of the temperature and the definition of the absolute temperature scale, as well as the definition of a thermodynamic system and a system formed by the product of two thermodynamic systems. In addition, he also provided new formulations of the first and second laws of thermodynamics, introduced the concept of the accumulation function, and described how it operates in thermal processes for cycles. It was also demonstrated that the first law for a cycle implies an inequality for the work accomplished in it, which involves an integral similar to that of Clausius [9]. Serrin’s work in thermodynamics offers a perspective different from what is typically found in the literature and in classical thermodynamics courses for science and engineering.

Ideal thermal cycles are fundamental in science and engineering, as they allow for the analysis of energy conversion systems that serve as the basis for engine design [1]. Within Serrin’s thermodynamic framework, the accumulation function has been obtained for the ideal Otto, Diesel, Stirling, and Ericsson cycles [9].

The ideal Brayton cycle has been widely studied within the classical thermodynamic framework, which includes equilibrium states and reversible processes [2]. This has extended to the introduction of an irreversibility factor and a time factor to simulate the primary heat-transfer processes for the rate of energy exchange between the heat engine and its surroundings, thereby defining the endoreversible Brayton cycle [10] through finite-time thermodynamics (FTT), as proposed by Curzon and Ahlborn to study a Carnot engine with external irreversibilities [11] and internal irreversibilities in the Brayton cycle [12].

Studies have examined the relationship between entropy production and thermal efficiency or net work in the design of gas turbine engines [13] as well as the use of the thermoeconomic approach to optimize the thermoeconomic objective function of an irreversible regenerative Brayton refrigeration cycle [14], modifications through the Maisotsenko–Brayton cycle model [15,16], a thermodynamic model of a modified closed binary Brayton cycle (MCBBC) [17], and electrochemical models for the case of a thermally regenerative electrochemical refrigerator (TRER) [18]. Nuclear, solar, and experimental applications [19], including waste-heat recovery through the use of thermodynamic cycles for power generation [20], have also been explored.

A theoretical model of a combined regenerative Brayton cycle+open inverse Brayton cycle (OCBC) within the framework of finite-time thermodynamics (FTT) [21] has been proposed. The model of the supercritical CO_2_ Brayton cycle (RBC-PC) [22], and the composite configuration RBC-PC-IHR [23], as well as a finite-time thermodynamic (FTT) model for a regenerative supercritical CO_2_ Brayton cycle, and studies in recompression configurations, such as a bottoming cycle coupled to a marine gas turbine [24], and an endoreversible model of a modified simple closed Brayton cycle (MESCBC) [25], have also been examined.

Nevertheless, the ideal Brayton cycle has not been studied within Serrin’s thermodynamic framework, and the accumulation function has not been derived for this thermal cycle.

In Section 2, an alternative mathematical development is presented to calculate the inequality for the work accomplished in a cycle, following Serrin and Huilgol’s theoretical framework [9,26,27,28,29], and explicitly introducing the temperature ranges in which the system exchanges heat are finite; i.e., there are two extreme temperatures that bound the interval during which heat exchanges occur between the system and its surroundings. This clearly shows the natural physical fact that heat exchange processes in a system have two defined extreme temperatures, corresponding to the beginning and end of the process, which can be equal in the isothermal limiting case.

In Section 3, the derivation of the accumulation function for the ideal air-standard Brayton cycle is presented, where the temperature constraints $T_{2}$ and $T_{4}$ of the adiabatic compression and expansion processes under which it operates are analyzed. Subsequently, a practical example of the accumulation function for this cycle is provided along with its graphical representation.

Finally, in Section 4, the conclusions are presented.

## 2. Work Done in a Thermodynamic Cycle

The inequality for the work that takes place during a thermodynamic cycle is calculated, as obtained by Serrin [26], with the difference that this case is based on bounding the temperature range within which the heat absorption and rejection processes occur in the system.

Following Huilgol and Serrin [9,26,27], consider a thermodynamic system S. As examples, consider a gas body or an elastic solid. Such a system S is endowed with a set of processes P(S) that it can undergo, which are denoted by *P*, *R*, *S*, etc. Moreover, the set P(S) of processes has a subset $P_{c}\left(S\right)$ of reversible and irreversible cyclic processes.

A reversible system S is endowed with a finite-dimensional state space Σ, which is an open and connected subset of $R^{n}$. The space Σ denotes the set of possible macroscopic states of the system, described by a finite number of independent state variables, such as pressure and volume. For each process P∈P(S), there exists a corresponding unique path Γ associated with Σ, such that Γ:I→Σ, where I = [a,b] denotes a closed time interval, and the function t→Γ(t) is differentiable. In particular, the path of any cyclic process is assumed to be closed, i.e., Γ(a)=Γ(b). Additionally, it is assumed that there exist two differential forms dw and dq defined on Σ with continuous coefficients associated with infinitesimal changes in energy transfer. These two expressions have the property of yielding the work accomplished and the heat transferred in a process, and they operate as follows:

(1) $$\bar{W}\left(P\right)\;=\;\int_{\Gamma }dw,\;\bar{Q}\left(P\right)\;=\;\int_{\Gamma }dq$$

where Γ is the path associated with the process *P*, and the convention is adopted that if $\bar{W}\left(P\right)>0$, the thermodynamic system does work on the surroundings, and if $\bar{Q}\left(P\right)>0$, the working substance absorbs heat from the surroundings. Additionally, the work accomplished in a cycle and the heat transferred in a cycle are denoted by $W_{c}$ and $Q_{c}$, respectively.

The first law for any cyclic process of a thermodynamic system takes the form $W_{c}=Q_{c}$ [9], since the system returns to its initial state at the end of the cycle and the total change in internal energy is zero [2]. The accumulation function is defined by Serrin as the difference between the heat absorbed by the working substance in a cyclic process, $Q_{c}^{+}\geq 0$, and the heat rejected during the cycle, $Q_{c}^{-}\geq 0$ [26]. That is, $Q_{c}=Q_{c}^{+}-Q_{c}^{-}$, so that

(2) $$W_{c}=Q_{c}^{+}-Q_{c}^{-}$$

Now, consider a regular thermodynamic closed cycle that receives heat from a high-temperature thermal reservoir, $Q_{c}^{+}>0$, and rejects heat to a low-temperature thermal reservoir, $Q_{c}^{-}>0$, and the finite time interval I=[0,1] of a cycle denoted by $I\left(t\right)=I_{c}^{+}\left(t\right)\cup I_{c}^{-}\left(t\right)\cup I_{c}^{0}\left(t\right)$ with 0≤t≤1.

The interval $I_{c}^{+}\left(t\right)$ is defined as the time interval during which the heat transfer rate is positive, $\dot{q}\left(t\right)>0$; i.e., a positive amount of heat is absorbed by the cycle. Subsequently, the interval $I_{c}^{-}\left(t\right)$ is defined as the time interval during which heat is rejected by the cycle, and therefore $\dot{q}\left(t\right)<0$. Finally, the interval $I_{c}^{0}\left(t\right)$ is defined for an adiabatic process where $\dot{q}\left(t\right)=0$. According to Huilgol [30],

(3) $$Q_{c}^{+}=\int_{I_{c}^{+}\left(t\right)}\dot{q}\left(t\right)dt$$

is defined as the heat absorbed by a body and

(4) $$Q_{c}^{-}=\int_{I_{c}^{-}\left(t\right)}[-\dot{q}\left(t\right)]dt$$

is the heat rejected by the body. Thenceforth, Diaz used two constant temperatures at the beginning and end of each process that undergoes a thermodynamic cycle [31,32]. This means that during the energy transfer process occurring in the interval $I_{c}^{+}\left(t\right)$, there are minimum and maximum constant temperatures, i.e., $0<T_{min}^{+}\leq T\left(t\right)\leq T_{max}^{+}<\infty$, ensuring that the temperature during this interval is bounded, i.e., the temperature range in which the energy exchange process takes place must be finite, and the extreme temperature values of the interval correspond to the start and end of the process, being equal in the isothermal limiting case.

It is noted that in practice, the method for approximating the value of these temperatures depends on the cycle under which the engine of interest operates. For example, in a gas turbine, the final temperature reached by the system during heat rejection is considered to be equal to the ambient temperature, while the maximum temperature of the cycle is limited by the properties of the construction materials [13]. The other two important temperatures can be approximated using the adiabatic equations of the ideal air-standard Brayton cycle or through tables of the thermodynamic properties of air [4].

Now, considering the inverse, which is also bounded:

(5) $$\frac{1}{T_{min}^{+}}\geq \frac{1}{T(t)}\geq \frac{1}{T_{max}^{+}}$$

Multiplying (5) by $\dot{q}\left(t\right)$, which is positive, and integrating over $I_{c}^{+}\left(t\right)$, it follows that

(6) $$\frac{Q_{c}^{+}}{T_{min}^{+}}\geq \int_{I_{c}^{+}\left(t\right)}\frac{\dot{q}\left(t\right)}{T(t)}dt\geq \frac{Q_{c}^{+}}{T_{max}^{+}}$$

where Equation (3) has been considered, and $T_{min}^{+}$ and $T_{max}^{+}$ are constants. Similarly, for the interval $I_{c}^{-}\left(t\right)$ with $\dot{q}\left(t\right)<0$, there are two constant temperatures such that $0<T_{min}^{-}\leq T\left(t\right)\leq T_{max}^{-}<\infty$, and it follows that

(7) $$-\frac{Q_{c}^{-}}{T_{min}^{+}}\leq -\int_{I_{c}^{-}\left(t\right)}\frac{\left[-\dot{q}\left(t\right)\right]}{T(t)}dt\leq -\frac{Q_{c}^{-}}{T_{max}^{+}}$$

where Equation (4) has been considered. Next, adding (6) and (7) and taking into account that

(8) $$\oint_{I_{c}\left(t\right)}\frac{\dot{q}\left(t\right)}{T(t)}dt=\int_{I_{c}^{+}}\frac{\dot{q}\left(t\right)}{T(t)}dt-\int_{I_{c}^{-}}\frac{\dot{q}\left(t\right)}{T(t)}dt$$

Since over $I_{c}^{0}$ there is an adiabatic process and $\dot{q}\left(t\right)=0$, it follows that

(9) $$\frac{Q_{c}^{+}}{T_{max}^{+}}-\frac{Q_{c}^{-}}{T_{min}^{+}}\leq \oint_{I_{c}\left(t\right)}\frac{\dot{q}\left(t\right)}{T(t)}dt\leq \frac{Q_{c}^{+}}{T_{min}^{+}}-\frac{Q_{c}^{-}}{T_{max}^{+}}$$

Now, solving Equation (2) for $Q_{c}^{-}$ and substituting into

(10) $$\frac{Q_{c}^{+}}{T_{max}^{+}}-\frac{Q_{c}^{-}}{T_{min}^{+}}\leq \oint_{I_{c}\left(t\right)}\frac{\dot{q}\left(t\right)}{T(t)}dt$$

It follows that

(11) $$W_{c}\leq \left[1-\frac{T_{min}^{-}}{T_{max}^{+}}\right]Q_{c}^{+}+T_{min}^{-}\oint_{I_{c}\left(t\right)}\frac{\dot{q}\left(t\right)}{T(t)}dt$$

On the other hand, using $Q_{c}^{-}$ in

(12) $$\oint_{I_{c}\left(t\right)}\frac{\dot{q}\left(t\right)}{T(t)}dt\leq \frac{Q_{c}^{+}}{T_{min}^{+}}-\frac{Q_{c}^{-}}{T_{max}^{+}}$$

It follows that

(13) $$\left[1-\frac{T_{max}^{-}}{T_{min}^{+}}\right]Q_{c}^{+}+T_{max}^{-}\oint_{I_{c}\left(t\right)}\frac{\dot{q}\left(t\right)}{T(t)}dt\leq W_{c}$$

and, from Equations (11) and (13), the expression for the first law of thermodynamics derived by Huilgol is obtained [9]:

(14) $$\left[1-\frac{T_{max}^{-}}{T_{min}^{+}}\right]Q_{c}^{+}+T_{max}^{-}\oint_{I_{c}\left(t\right)}\frac{\dot{q}\left(t\right)}{T(t)}dt\leq W_{c}\leq \left[1-\frac{T_{min}^{-}}{T_{max}^{+}}\right]Q_{c}^{+}+T_{min}^{-}\oint_{I_{c}\left(t\right)}\frac{\dot{q}\left(t\right)}{T(t)}dt$$

The above demonstrates that the first law, as stated by Serrin for cycles, implies an inequality involving the minimum and maximum temperatures of the processes experienced by the system during a cycle, as well as the Clausius integral. In other words, the first law anticipates, so to speak, the importance of this integral in the second law, as pointed out by Huilgol [9], since it defines the absolute temperature scale.

## 3. The Accumulation Function for the Ideal Air-Standard Brayton Cycle

The ideal Brayton cycle is a theoretical thermodynamic cycle in which a working fluid undergoes four reversible processes cyclically [4]. When the working fluid is air, this thermodynamic cycle is also known as the ideal air-standard Brayton cycle and represents the ideal operating processes of a gas turbine [2]. This cycle operates under the following two main idealizations [3]:

1. The air behaves as an ideal gas;
2. Energy is supplied to the system through heat transfer from a high-temperature thermal reservoir, which is maintained at a constant temperature.

Under these two assumptions, the properties $C_{p}$ and $C_{V}$, referred to as specific heats, remain constant [4], meaning that

(15) $$\gamma =\frac{C_{p}}{C_{V}}>1$$

where the property γ is the specific heat ratio. Furthermore, effects on the system due to kinetic and potential energy are neglected; likewise, irreversibilities such as pressure drops due to friction or heat losses to the surroundings are neglected [2].

The processes experienced by the working fluid during a complete cycle are described below [1,2,3,4]:

1–2 Adiabatic compression: Work is performed on the air as it transitions from state 1 to state 2, decreasing its volume and increasing its pressure, with no heat exchange. The relationship between the temperatures and pressures of these states is given by the following adiabatic equation:
  (16) $$T_{1}P_{1}^{\frac{1-\gamma }{\gamma }}=T_{2}P_{2}^{\frac{1-\gamma }{\gamma }}$$

2–3 Isobaric heating: Heat is transferred to the compressed air at high pressure as it transitions from state 2 to state 3, increasing its temperature and volume;

3–4 Adiabatic expansion: The high-temperature, high-pressure air expands, performing work and decreasing its temperature and pressure with no heat exchange. The adiabatic equation that relates these two states is
  (17) $$T_{4}P_{1}^{\frac{1-\gamma }{\gamma }}=T_{3}P_{2}^{\frac{1-\gamma }{\gamma }}$$

4–1 Isobaric cooling: The low-pressure expanded air transitions from state 4 to state 1 while rejecting heat to the low-temperature reservoir, which is maintained at a constant temperature, reducing its volume and temperature, and returning to the initial condition of the cycle.

It is worth noting that although the idealizations of the ideal Brayton cycle allow it to be fully studied, they impose limits on the model for describing real Brayton cycle engines. The reversibility of the processes requires them to occur over infinitely long times, leading to zero power output.

Furthermore, in real Brayton cycle engines, there are heat losses to the environment, heat losses through thermal bridges, friction, and pressure drops. The heat transfer to the system is obtained from the combustion of an air–fuel mixture, rather than from a thermal reservoir at constant temperature, whereas the discharge of the combustion gases to the environment corresponds to the system’s heat rejection process [12,19,20]. Finally, although $C_{p}$ and $C_{V}$ can be used as constants in real Brayton cycle engines, this approximation introduces errors at high temperatures and pressures because their values increase as temperature and pressure rises and are not constant [2].

Now, to obtain a graphical representation of the processes described above that make up the ideal air-standard Brayton cycle in a p-V diagram, the temperatures $T_{1}=290$ K and $T_{3}=1100$ K are considered [10] as well as the constant pressure during the heat rejection process $p_{1}=101.325$ kPa and the universal gas constant R=8.314 J/mol K. For air, the specific heat ratio can be approximated as γ=1.4, and it is convenient to take n=1 mol of air, since this choice only sets the scale for volumes without altering the shape of the cycle [2,13]. From these values, the corresponding pressure ratio for the maximum work of the ideal air-standard Brayton cycle is shown below [2]:

(18) $$r_{opt}={\left(\frac{T_{3}}{T_{1}}\right)}^{\frac{\gamma }{2\left(\gamma -1\right)}}=10.3095$$

**Figure 1:**
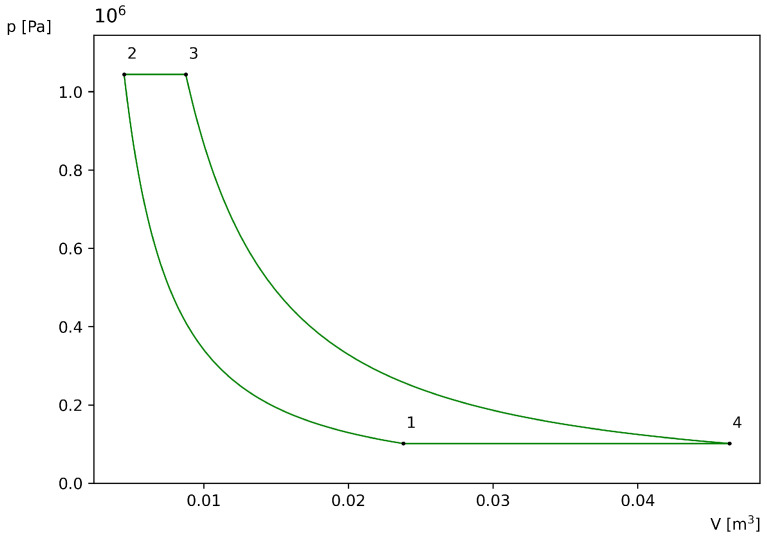
p-V diagram of the ideal air-standard Brayton cycle with $T_{4}=T_{2}=T_{2opt}=564.8008$ K, $p_{2}=1,044,617.5433$ Pa, $V_{1}=0.0237$ m^3^, $V_{2}=0.0044$ m^3^, $V_{3}=0.0087$ m^3^ and $V_{4}=0.0463$ m^3^.

Using these values in the ideal gas equation (pV=nRT) and Equations (16) and (17), which correspond to the adiabatic compression and expansion processes, the curves shown in Figure 1 are obtained.

To quantify the influence of assuming constant values for the numerical calculation, it is first noted that at 250 K, γ=1.401 and at 300 K, γ=1.400 [4]. Thus, the variation in this interval is Δγ=0.001, which corresponds to a relative error of |Δγ|/γ≈0.07%. Therefore, using γ=1.4 at $T_{1}=290$ K does not introduce significant errors.

Next, by implicitly differentiating Equation (18) to estimate the error associated with assuming constant γ,

(19) $$\frac{\Delta r}{r}\approx -\frac{\ln\frac{T_{3}}{T_{1}}}{2{\left(\gamma -1\right)}^{2}}\Delta \gamma$$

using the values employed in Figure 1, this gives Δr/r≈−4.1662Δγ. At 1100 K, the value of γ is 1.336 [4], and it is obtained that Δγ=−0.064. Consequently, Equation (19) yields a relative error of Δr/r≈26.66%, which is consistent with the variation of $C_{p}\left(T\right)$ in the range 290–1100 K [4].

Therefore, the deviations resulting from using constant values in the determination of $T_{opt}$ and in the calculation of $T_{2}$ and $T_{4}$ in the example case, as well as in the point coordinates of $W_{c}$ and $Q_{c}^{+}$ do not significantly affect the results obtained.

### 3.1. Calculation of the Accumulation Function

In the non-adiabatic processes of the ideal air-standard Brayton cycle, it is possible to determine the amount of heat transferred as a function of temperature. However, during the adiabatic processes, such a relationship is difficult to determine because the heat transferred is zero. Specifically, in adiabatic compression, no heat is transferred while the temperature increases; similarly, in adiabatic expansion, the temperature decreases without any heat exchange. The accumulation function serves as a tool to resolve this, as it makes explicit, in each process, the amount of heat transferred up to and including any temperature at any point of the process [9]. Nevertheless, this does not replace the standard γ-based expressions, which are fundamental to the description of adiabatic processes.

To determine the accumulation function of the ideal air-standard Brayton cycle, the absolute temperature scale *T* is considered. Subsequently, when subtracting Equations (16) and (17), the following is obtained:

(20) $$\left(T_{4}-T_{1}\right)p_{1}^{\frac{1-\gamma }{\gamma }}=\left(T_{3}-T_{2}\right)p_{2}^{\frac{1-\gamma }{\gamma }}$$

By rearranging, an equation can be obtained that relates the four temperatures and the two important pressures present in a complete cycle:

(21) $$\frac{T_{3}-T_{2}}{T_{4}-T_{1}}={\left(\frac{p_{1}}{p_{2}}\right)}^{\frac{1-\gamma }{\gamma }}$$

Since $p_{2}>p_{1}$, $T_{2}<T_{3}$, $T_{1}<T_{4}$, $T_{1}<T_{3}$, and considering Equation (15) as well as the pressure ratio of the process 1-2, $r=p_{2}/p_{1}>1$, it can be deduced that $T_{3}-T_{2}>T_{4}-T_{1}$.

Now, it is necessary to specify how the values of $T_{2}$ compare with $T_{4}$. To obtain a relationship between these two temperatures, Equations (16) and (17) can be used again, and it is found that for $T_{2}>T_{4}$ to hold, the following condition must be satisfied:

(22) $$r>{\left(\frac{T_{3}}{T_{1}}\right)}^{\frac{\gamma }{2\left(\gamma -1\right)}}$$

That is, $T_{2}$ will be greater than $T_{4}$ whenever Equation (22) holds, and by comparing this result with Equation (18), it follows that $r>r_{opt}$, which means that the cycle operates with a pressure ratio higher than the optimal one.

Next, during the isobaric heating process corresponding to the transition from state 2 to state 3, the accumulation function must ensure that the heat absorption process occurs at constant pressure, p=constant. Thus,

(23) $$Q^{+}\left(P,T\right)=\left\{\begin{array}{cc} 0 & T<T_{2},\\ C_{p}\left(T-T_{2}\right) & T_{2}\leq T<T_{3},\\ C_{p}\left(T_{3}-T_{2}\right) & T_{3}\leq T<\infty . \end{array}\right.$$

And employing the Heaviside function, which works as follows H(x)=0, x<0, H(x)=1, x≥1, the following is obtained:

(24) $$Q^{+}\left(P,T\right)=C_{p}\left(T-T_{2}\right)H\left(T-T_{2}\right)-C_{p}\left(T-T_{3}\right)H\left(T-T_{3}\right)$$

For 0<T<∞. Similarly, during process 4-1, the heat rejection process occurs at p=constant, yielding

(25) $$Q^{-}\left(P,T\right)=\left\{\begin{array}{cc} 0 & T<T_{1},\\ C_{p}\left(T-T_{1}\right) & T_{1}\leq T<T_{4},\\ C_{p}\left(T_{4}-T_{1}\right) & T_{4}\leq T<\infty . \end{array}\right.$$

And using the Heaviside function:

(26) $$Q^{-}\left(P,T\right)=C_{p}\left(T-T_{1}\right)H\left(T-T_{1}\right)-C_{p}\left(T-T_{4}\right)H\left(T-T_{4}\right)$$

For 0<T<∞. Consequently, over a complete cycle, it follows that

(27) $$Q_{c}\left(T\right)=C_{p}\left(T_{3}-T_{2}\right)-C_{p}\left(T_{4}-T_{1}\right)$$

For 0<T<∞.

Therefore, the accumulation function over the positive real axis is

(28) $$Q\left(P,T\right)=\left\{\begin{array}{cc} 0 & T<T_{1},\\ -C_{p}\left(T-T_{1}\right) & T_{1}\leq T<T_{4},\\ -C_{p}\left(T_{4}-T_{1}\right) & T_{4}\leq T<T_{2},\\ C_{p}\left(T-T_{2}\right)-C_{p}\left(T_{4}-T_{1}\right) & T_{2}\leq T<T_{3},\\ C_{p}\left(T_{3}-T_{2}\right)-C_{p}\left(T_{4}-T_{1}\right) & T_{3}\leq T<\infty . \end{array}\right.$$

Equation (28) mathematically describes the variation of heat as a function of temperature during the processes of the ideal air-standard Brayton cycle. For temperature values lower than $T_{1}$, no heat has been exchanged in the system. In the interval $T_{1}\leq T<T_{4}$, which corresponds to the isobaric heat rejection process to a low-temperature reservoir, heat is rejected linearly, as the amount of heat decreases proportionally with the decrease in temperature ($\partial Q/\partial T=-C_{p}$). In the interval $T_{2}\leq T<T_{3}$, corresponding to the isobaric heat addition process to the system from a high-temperature reservoir, heat is supplied linearly, as the amount of heat increases proportionally with the increase in temperature ($\partial Q/\partial T=C_{p}$). In the interval $T_{4}\leq T<T_{2}$ and for temperature values higher than $T_{3}$, the system does not exchange heat, and the function $Q\left(P,T\right)$ remains constant and equal to the total amount of heat exchanged during a cycle for temperatures greater than $T_{3}$; therefore, ∂Q/∂T=0.

The sensitivity of Q(P,T) with respect to $T_{1}$ and $T_{3}$ is $\partial Q/\partial T_{l}=C_{p}$, l=1,3, in the temperature intervals where these variables appear explicitly and zero otherwise. Thus, increasing $T_{1}$ increases Q(P,T) for all temperatures $T\geq T_{1}$, whereas increasing $T_{3}$ increases Q(P,T) only in the last interval ($T\geq T_{3}$). Similarly, for $T_{2}$ and $T_{4}$, the sensitivity in the intervals where they appear explicitly is $\partial Q/\partial T_{j}=-C_{p}$, j=2,4, and it is zero in all other intervals. Consequently, increasing $T_{2}$ and $T_{4}$ decreases Q(P,T) in those intervals that depend explicitly on $T_{2}$ and in those where the term $T_{4}-T_{1}$ appears.

The sensitivity of Q(P,T) with respect to $C_{p}$ follows directly from Equation (28). In the interval $T_{1}\leq T<T_{4}$, the sensitivity is negative and its magnitude increases monotonically with $T-T_{1}$. For $T_{4}\leq T<T_{2}$, the derivative is a constant negative value, $\partial Q/\partial C_{p}=-\left(T_{4}-T_{1}\right)$, indicating that Q(P,T) decreases linearly with increasing $C_{p}$. In the interval $T_{2}\leq T<T_{3}$, the sign of the sensitivity depends on the relationship between $T-T_{2}$ and $T_{4}-T_{1}$. The transition temperature at which $T-T_{2}$ exceeds $T_{4}-T_{1}$ is $T_{*}=T_{2}+\left(T_{4}-T_{1}\right)$. At $T=T_{*}$, the derivative becomes zero, while for $T>T_{*}$, it is positive, implying that Q(P,T) increases with $C_{p}$. For $T\geq T_{3}$, a positive derivative is obtained, since—as already derived from Equation (21)—the condition $T_{3}-T_{2}>T_{4}-T_{1}$ holds; therefore, Q(P,T) increases linearly with $C_{p}$ in this temperature range.

In the limiting case where $T_{4}\to T_{2}$, Equation (28) takes the following form:

(29) $$Q_{T_{4}\to T_{2}}\left(P,T\right)=\left\{\begin{array}{cc} 0 & T<T_{1},\\ -C_{p}\left(T-T_{1}\right) & T_{1}\leq T<T_{2opt},\\ C_{p}\left(T+T_{1}-2T_{2opt}\right) & T_{2opt}\leq T<T_{3},\\ C_{p}\left(T_{3}+T_{1}-2T_{2opt}\right) & T_{3}\leq T<\infty . \end{array}\right.$$

where $T_{2opt}$ corresponds to the temperature at which the maximum work output is achieved in the ideal air-standard Brayton cycle.

To verify that Equation (28) is correct, it must satisfy Serrin’s Accumulation Theorem and Clausius’ integral for a cycle. First, from the Accumulation Theorem, it follows that

(30) $$\begin{array}{cc} \int_{0}^{\infty }\frac{Q\left(P,T\right)}{T^{2}}dT= & \int_{T_{1}}^{T_{4}}\frac{-C_{p}\left(T-T_{1}\right)}{T^{2}}dT+\int_{T_{4}}^{T_{2}}\frac{-C_{p}\left(T_{4}-T_{1}\right)}{T^{2}}dT+\\ & \int_{T_{2}}^{T_{3}}\frac{C_{p}\left(T-T_{2}\right)-C_{p}\left(T_{4}-T_{1}\right)}{T^{2}}dT+\int_{T_{3}}^{\infty }\frac{C_{p}\left(T_{3}-T_{2}\right)-C_{p}\left(T_{4}-T_{1}\right)}{T^{2}}dT \end{array}$$

and, by solving each integral, the following is obtained:

(31) $$\begin{array}{cc} \int_{0}^{\infty }\frac{Q\left(P,T\right)}{T^{2}}dT= & C_{p}\{-\ln\frac{T_{4}}{T_{1}}+\ln\frac{T_{3}}{T_{2}}-T_{1}\left(\frac{1}{T_{4}}-\frac{1}{T_{1}}\right)+T_{2}\left(\frac{1}{T_{3}}-\frac{1}{T_{2}}\right)+\\ & \left(T_{4}-T_{1}\right)\left(\frac{1}{T_{2}}-\frac{1}{T_{4}}+\frac{1}{T_{3}}-\frac{1}{T_{2}}\right)+[\left(T_{3}-T_{2}\right)-\left(T_{4}-T_{1}\right)]\left(\lim_{t\to \infty }\frac{-1}{t}+\frac{1}{T_{3}}\right)\} \end{array}$$

and eliminating similar terms, the expression simplifies to

(32) $$\int_{0}^{\infty }\frac{Q\left(P,T\right)}{T^{2}}dT=C_{p}\ln\left(\frac{T_{1}T_{3}}{T_{2}T_{4}}\right)$$

However, from Equations (16) and (17), it follows that the four temperatures satisfy the following condition:

(33) $$\frac{T_{1}T_{3}}{T_{2}T_{4}}=1$$

Then, using Equation (33) in Equation (32), it is found that the function Q satisfies the integral from the Accumulation Theorem, namely:

(34) $$\int_{0}^{\infty }\frac{Q\left(P,T\right)}{T^{2}}dT=0$$

which corresponds to a reversible cycle.

Finally, for Clausius’ integral, it follows that over a complete cycle,

(35) $$\oint \frac{dQ\left(P,T\right)}{T}=C_{p}\int_{T_{2}}^{T_{3}}\frac{dT}{T}-C_{p}\int_{T_{1}}^{T_{4}}\frac{dT}{T}=C_{p}\ln\left(\frac{T_{1}T_{3}}{T_{2}T_{4}}\right)$$

And from Equation (33), it follows that

(36) $$\oint \frac{Q\left(P,T\right)}{T}dT=0$$

Thus, *Q* also satisfies Clausius’ integral for a complete cycle.

Now, from the direct application of Equation (14) to the ideal air-standard Brayton cycle, the following expression is obtained:

(37) $$\left(1-\frac{T_{4}}{T_{2}}\right)\leq \frac{W_{c}}{Q_{c}^{+}}\leq \left(1-\frac{T_{1}}{T_{3}}\right)$$

The actual energy efficiency of the ideal air-standard Brayton cycle, using the relationship given by Equation (33), can be expressed as follows [2]:

(38) $$\eta =1-\frac{T_{4}}{T_{3}}$$

The upper limit in Equation (37) corresponds to the maximum efficiency of a Carnot engine operating between two thermal reservoirs at temperatures $T_{1}$ and $T_{3}$. This efficiency is greater than the actual efficiency of the ideal air-standard Brayton cycle given by Equation (38), since $T_{1}<T_{4}$. On the other hand, the term on the left-hand side of the inequality in Equation (37) represents a theoretical lower bound as a function of the final temperatures of the adiabatic processes. To maintain the inequality in Equation (37), and taking into account Equation (38), the condition $T_{4}\leq T_{2}$ must be satisfied. Thus, the range of physically realizable values in Equation (37) lies within the interval [0,1), since, as $T_{4}$ approaches $T_{2}$, this lower bound decreases to zero in the case of equality.

### 3.2. Example Case

To illustrate the behavior of the previously obtained accumulation function of the ideal air-standard Brayton cycle and analyze how it describes the behavior of the exchanged heat and explicitly shows the key temperatures over a complete cycle, consider the same values used in Figure 1, namely, $T_{1}=290$ K; $T_{3}=1100$ K; $P_{1}=101.325$ kPa; the universal gas constant R=8.314 J/molK; γ=1.4; and n=1 mol of air. Here, $T_{1}$ corresponds to the final ambient temperature reached by the system during heat rejection at ambient pressure.

**Figure 2:**
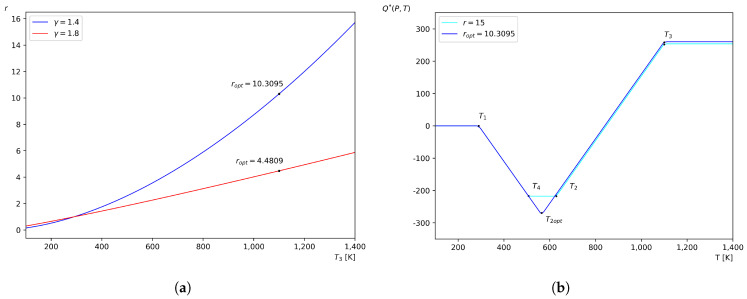
Dependence of the pressure ratio *r* with $T_{3}$ (**a**), and the accumulation function *Q* for the example case of the ideal air-standard Brayton cycle (**b**).

Now, the value of $T_{4}$ relative to $T_{2}$ must be considered. Figure 2a shows the behavior of *r* as a function of $T_{3}$, in accordance with Equation (22), for two different values of γ and the respective optimum value for maximum work.

Then, since for these values, it was obtained that $r_{opt}=10.3095$ in Equation (18), to satisfy Equation (22), a value of r=15 is taken for convenience [21]. This results in $p_{2}=1519875.0$ Pa, $T_{2}=628.6719$ K, $T_{4}=507.4188$ K, $V_{1}=0.0237$ m^3^, $V_{2}=0.0034$ m^3^, $V_{3}=0.00601$ m^3^, and $V_{4}=0.0416$ m^3^. Equation (33) for these temperatures yields $T_{1}T_{3}/T_{2}T_{4}=290\times 1100/507.4188\times 628.6719=1.0000$ and $T_{1}T_{3}/T_{2opt}T_{2opt}=290\times 1100/564.808\times 564.808=1.0000$. Therefore, they satisfy Equation (32).

Using these values in Equation (28), and defining $Q^{*}\left(P,T\right)=Q\left(P,T\right)/C_{p}$ for simplicity of representation, the following is obtained:

(39) $$Q^{*}\left(P,T\right)=\left\{\begin{array}{cc} 0 & T<290\;K,\\ -T+290\;K & 290\;K\leq T<507.4188\;K,\\ -217.4188\;K & 507.4188\;K\leq T<628.6719\;K,\\ T-846.0908\;K & 628.6719\;K\leq T<1100\;K,\\ 253.9091\;K & 1100\;K\leq T<\infty . \end{array}\right.$$

Whereas, for the limiting case given by Equation (29), where $T_{4}\to T_{2}$, the expression is

(40) $$Q_{T_{4}\to T_{2}}^{*}\left(P,T\right)=\left\{\begin{array}{cc} 0 & T<290\;K,\\ -T+290\;K & 290\;K\leq T<564.8008\;K,\\ T-839.6016\;K & 564.8008\;K\leq T<1100\;K,\\ 260.3983\;K & 1100\;K\leq T<\infty . \end{array}\right.$$

where it has been defined that $Q_{T_{4}\to T_{2}}^{*}\left(P,T\right)=Q_{T_{4}\to T_{2}}\left(P,T\right)/C_{p}$ for simplicity.

The behavior of Equations (39) and (40), *Q* as a function of *T*, is shown in Figure 2b. On the one hand, for Equation (39), it can be observed that in the interval $T_{1}\leq T<T_{4}$, which corresponds to isobaric cooling, heat is rejected linearly from the system; the amount of heat decreases proportionally with the decrease in temperature. On the other hand, for Equation (40), isobaric cooling falls within the interval $T_{1}\leq T<T_{2opt}$. In the interval $T_{2}\;\mathrm{or}\;T_{2opt}\leq T<T_{3}$, corresponding to isobaric heating, heat is supplied linearly to the system; the amount of heat increases proportionally with the increase in temperature. In the other temperature ranges, the function Q remains constant, since there is no further heat exchange in the system.

In Figure 2a, it can be observed that as the value of γ increases, the value of $r_{opt}$ decreases. On the other hand, in Figure 2b, it can be seen that increasing the value of *r*, and consequently the values of $T_{4}$ and $T_{2}$, reduces the value of *Q* after heat rejection, as well as the value of *Q* during the heat absorption process. This results in a decrease in the constant value of *Q* in the total amount of heat exchanged. In contrast, increasing the values of $T_{1}$ and $T_{3}$ leads to an increase in the value of *Q*.

**Figure 3:**
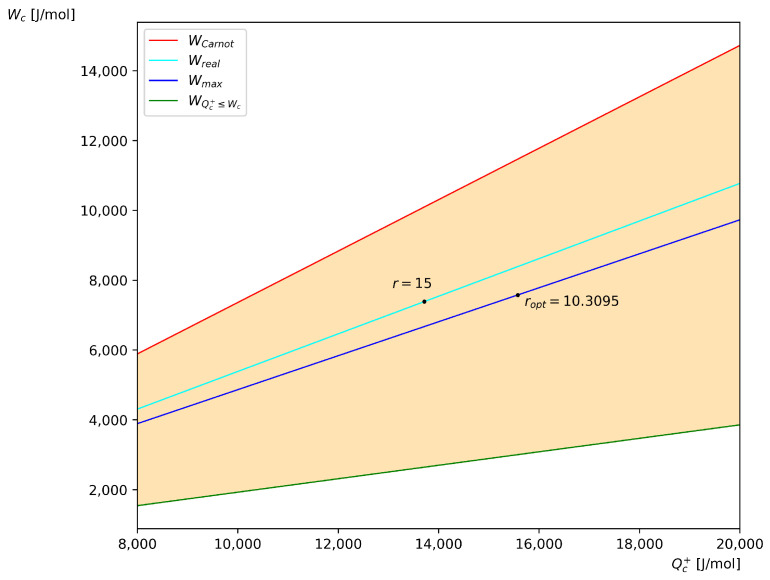
Work curves as a function of $Q_{c}^{+}$ for the ideal air-standard Brayton cycle with $C_{p}=29.1$ J/mol K.

On the other hand, Figure 3 shows the work curves as a function of $Q_{c}^{+}$, which is in accordance with Equations (37) and (38). $W_{real}$ and $W_{max}$ are obtained from Equation (38) for the temperature values corresponding to r=15 and the optimum value $r_{opt}=10.3095$, respectively. $W_{Q_{c}^{+}\leq W_{c}}$ and $W_{Carnot}$ correspond to the lower and upper bounds of Equation (37) for the temperature values corresponding to r=15.

In Figure 3, the points r=15 and $r_{opt}=10.3095$ correspond to the coordinates (7388.759, 13,715.6477) and (7577.5919, 15,574.2967), respectively, i.e., the maximum achievable work is greater than the work obtained for a value larger than $r_{opt}$, as expected, although a greather amount of supplied heat is required.

## 4. Conclusions

In this work, the explicit form of the accumulation function for the ideal air-standard Brayton cycle has been presented based on Serrin’s thermodynamic framework. It has been shown how the four important temperatures in the cycle are compared, particularly the relationship that must be maintained for $T_{2}>T_{4}$, as well as the limiting case $T_{4}\to T_{2}$.

A more straightforward derivation than that made by Huilgol is presented to obtain the inequality for the work accomplished in a cycle, following the theoretical framework of Serrin and Huilgol and taking into account the restriction imposed by the accessible temperature range of the processes involved in the cycle. Within this framework, the first law yields a direct relationship between the heat exchanged and the temperatures at which it is transferred.

The accumulation function takes the form of a piecewise continuous function that allows determining the amount of heat transferred at any temperature during the cycle; for example, during the compression process, the temperature change causes energy accumulation in the system. This energy can be calculated using the accumulation function. Thus, with this expression, a continuous representation of the heat exchange behavior during each process of the cycle is obtained, clearly showing the key temperatures $T_{1}$, $T_{2}$, $T_{3}$, and $T_{4}$. This approach facilitates the physical interpretation of the heat transfer process and energy accumulation across the entire temperature range.

The example case quantitatively demonstrates how Serrin’s thermodynamic framework, applied to the ideal air-standard Brayton cycle, offers a different perspective from that typically studied in standard thermodynamics courses for science and engineering. It provides a clear reference for the transition temperatures between each process and helps to identify the global thermodynamic behavior of the cycle. For example, it was shown that increasing the values of temperatures $T_{2}$ and $T_{4}$ decreases the value of *Q*, resulting in less work produced compared to the maximum work generated for $r_{opt}$. However, this difference could be compensated by requiring a smaller amount of supplied heat.

This analysis could help identify which process of the cycle requires more energy for heating or cooling and determine trade-offs between the heat supplied to the system and the work performed.

Since the accumulation function of the Brayton cycle has been obtained for the ideal case, an accumulation function that accounts for irreversibilities would be more effective in specific applications. This can be accomplished by deriving an accumulation function for an endoreversible Brayton cycle, where finite thermal resistances at the hot and cold heat exchangers produce non-ideal heat-addition and heat-rejection processes. Alternatively, one could introduce pressure losses and non-ideal component efficiencies. Such developments would be more effective in practical applications.

## Data Availability

Data are contained within the article.
